# Influence of Macromolecular Architecture on the Optical and Humidity-Sensing Properties of Poly(*N*,*N*-Dimethylacrylamide)-Based Block Copolymers

**DOI:** 10.3390/polym10070769

**Published:** 2018-07-13

**Authors:** Katerina Lazarova, Marina Vasileva, Sijka Ivanova, Christo Novakov, Darinka Christova, Tsvetanka Babeva

**Affiliations:** 1 Institute of Optical Materials and Technologies, Bulgarian Academy of Sciences, 1113 Sofia, Bulgaria; marina@iomt.bas.bg; 2 Institute of Polymers, Bulgarian Academy of Sciences, 1113 Sofia, Bulgaria; sivanova@polymer.bas.bg (S.I.); hnovakov@polymer.bas.bg (C.N.); dchristo@polymer.bas.bg (D.C.)

**Keywords:** macromolecular architecture, poly(*N*,*N*-dimethylacrylamide) copolymers, thin films, humidity sensing, optical sensors

## Abstract

The influence of the macromolecular architecture of block copolymers containing poly(*N*,*N*-dimethyl acrylamide) (PDMA) on the optical characteristics and sensing properties of corresponding thin films is discussed. Series of hydrophilic PDMA-based copolymers of different chemical composition and chain architecture such as triblock, star-shaped, and branched were synthesized. The copolymers were characterized using conventional spectroscopic techniques as well as methods for characterization of copolymer macromolecular characteristics in solution, namely size-exclusion chromatography and static light scattering. Thin films of the copolymers of nanometer scale thickness were deposited on silicon substrates by the spin-coating method. The refractive index and extinction coefficient of the copolymer films were calculated from the reflectance spectra by using non-linear curve fitting methods and the composition-structure-optical properties relationships were evaluated. Humidity-sensing properties of the films were studied by measuring reflectance spectra of the films at a relative humidity range from 5 to 95%RH. The implementation of the copolymer films as optical sensors of humidity is justified and discussed.

## 1. Introduction

Complex macromolecular structures formed by covalently bonded blocks or material segments of different thermodynamic features are subject of immense research interest due to the unique properties in solution as well as at surface [1]. In this aspect, the macromolecular architecture can be used as an advanced tool in the attempts to tailor the copolymer properties for specific applications [2,3]. A particular example of emerging application is the optical sensing of humidity.

Humidity is present everywhere in our daily lives and it is our continuing companion. Moreover, humidity controls many environmental processes, because even at small variations in temperature, water evaporates or its vapors condense. At first glance it seems that humidity is harmless, but more in-depth analysis shows that in many cases humidity is undesirable and should be avoided. Humidity can damage sensitive materials and objects, disturb human comfort and negatively affect many manufacturing processes in food, pharmaceuticals and electronic industries. Thus, a demand for methods for precisely detecting and monitoring humidity is raised that explains the persistent scientific interest worldwide in this research field. Developing low cost materials through environmentally friendly methods of synthesis, on the one hand, and using simple methods of detection, on the other, will open new possibilities for sensitive and convenient detection of humidity with high resolution.

Due to some advantages over electronic sensors, such as room temperature operation, high accuracy, resistance to electromagnetic interference, and lack of explosion danger, optical sensing of humidity has gained increasing interest, and great research efforts have been made in recent years in this field [4,5]. The most common approach for optical sensing of humidity is using optical fibers covered with functional cladding sensitive to humidity [6]. Different materials have been used for functionalization of fibers, such as: Agarose gel [7]; graphene oxide [8]; reduced graphene oxide [9]; tungsten disulfide [10]; polyvinyl alcohol [11,12]; poly(*N*,*N*-dimethylacrylamide) (PDMA) [13]; and, bilayers of poly(allylamine hydrochloride) (PAH) and SiO_2_ mesoporous nanoparticles [14]. Despite the advantages of optical fiber sensors mentioned above, in addition to their small size and ability for remote sensing, their main drawbacks are related to the cost and complexity of optical equipment required for their operation. 

A relatively simpler alternative is color sensing of humidity where detection is performed by visual inspection of color, or color monitoring, by cheap camera [15,16,17]. Multilayered structures, called Bragg stacks, exhibiting structural colors, have already been used for humidity detection, where different thin film materials are used as sensitive media: Porous TiO_2_ deposited by glancing angle deposition method [18]; alternated tri-layers system of cellulose, poly(vinyl alcohol) and poly(*N*-vinylcarbazole) [19]; and, poly(2-hydroxylethyl methacrylate) and epoxy-based resist [20].

An even simpler approach for humidity detection is reported in [21,22], where polyacrylamide metallopolymers are utilized as sensing media and detection is performed by measuring the transmittance power with humidity variation. Depending on the particular metal complex used, different average sensitivities over a wide humidity range are obtained, with the highest reported value of 1.813 μW/%RH in the case of Zn (II) nitrate [22].

In our previous studies, we have demonstrated the humidity-sensing ability of thin films from poly(*N*,*N*-dimethyl acrylamide)-poly(ethylene oxide) (PDMA-PEO) di- and triblock copolymers deposited by spin coating on silicon substrates [23] or on top of a Bragg stack consisting of alternating layers of SiO_2_ and Nb_2_O_5_ [24]. Two detection methods were explored: Measuring reflectance for single films and transmittance for the stack, and visual inspection of color in both cases [23,24]. It was shown [23,24] that the optical response to humidity of the investigated copolymer films is little influenced by copolymer composition or structure (two- or tri-component; diblock or triblock). All studied copolymers exhibited high sensitivity toward humidity change from low to high levels. However, working with linear block copolymers of different composition and structure, we were not able to improve important sensing application parameters, such as hysteresis. Considering that the change in the measured signal is due to the swelling of the film when exposed to different humidity levels, it may be expected for the swelling to intensify when sophisticated polymer structures of hydrophilic nature and branched macromolecular architecture are employed for sensing.

In the present paper we study the influence of the macromolecular architecture of block copolymers containing PDMA on the optical characteristics and humidity-sensing properties of the corresponding thin films. Series of hydrophilic PDMA-based copolymers of different chemical composition and chain architecture such as linear (block) and branched are synthesized and characterized. The impact of macromolecular architecture on the optical and humidity-sensing properties of spin-on thin films is discussed and the implementation of polymer films for optical sensing of humidity is demonstrated.

## 2. Materials and Methods

### 2.1. Materials

The reagents used in the synthesis of polymers (monomer *N*,*N*-dimethyacrylamide (DMA); initiator ammonium cerium (IV) nitrate (NH_4_)_2_Ce(NO_3_)_6_; 1,1,1-tris(hydroxymethyl)propane (TMP); trimethylolpropane triacrylate (TMPTA); and, poly(ethylene glycol) diacrylate (PEGDA; av. *M*_n_ 575)) were purchased from Sigma-Aldrich (Steinheim, Germany). Poly(ethylene oxide) of molar mass 2000 g·mol^−1^ (PEO2000) was supplied from Fluka (Buchs, Switzerland). DMA was purified from inhibitor by passing through a column of basic alumina. All solvents and other reagents were of standard laboratory reagent grade and used as received. 

### 2.2. Polymer Synthesis

**Figure 1:**
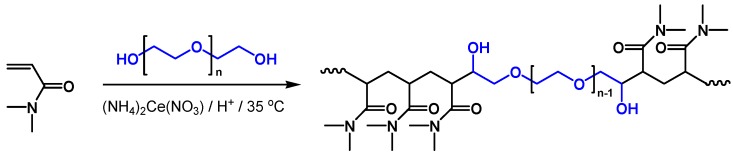
Reaction scheme of the synthesis of PDMA-*b*-PEO-*b*-PDMA triblock copolymer.

**Figure 2:**
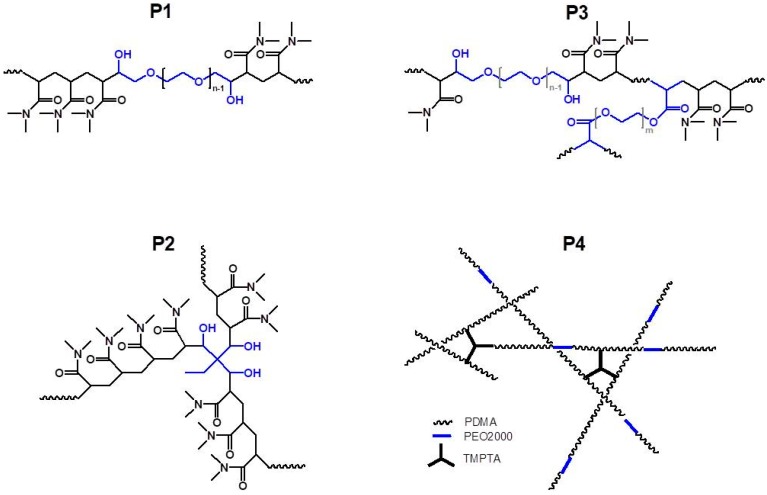
Schematic presentation of the structures of obtained copolymers P1–P4.

All copolymers studied in this work were synthesized by means of redox polymerization of DMA in deionized water using ammonium cerium (IV) nitrate as initiator and suitable hydroxyl functionalized initiating moiety. The reaction for obtaining triblock copolymer PDMA-*b*-PEO-*b*-PDMA (P1) was carried out as follows: A reaction mixture composed of DMA and PEO2000 (initial mole ratio 70:1; initial monomer concentration 4.2 g·L^−1^) in deionized water was prepared and bubbled with nitrogen for 15 min for oxygen removal. A solution of the initiator (NH_4_)_2_Ce(NO_3_)_6_ (in equimolar ratio to the hydroxyl end groups of the precursor) in 1 N HNO_3_ was added and the reaction mixture was bubbled with nitrogen for another 15 min. The polymerization was implemented for 3 h at 35 °C under nitrogen atmosphere. The reaction scheme of the synthesis is shown in Figure 1. The same procedure was applied to achieve tri-arm star-shaped PDMA (P2) when using TMP as a hydroxyl containing initiating moiety (initial mole ratio DMA:TMP = 45:1) (Figure 2). The obtained linear block copolymer, P1**,** and star-shaped polymer, P2, were purified by dialysis against deionized water and finally isolated by freeze-drying.

In order to synthesize branched copolymers of different macromolecular architecture, PEGDA (0.008 equiv. to the initial DMA concentration) or TMPTA (0.024 equiv. to the initial DMA concentration) were added to the initial reaction mixture following the same procedure as described above. The structures of synthesized branched copolymers P3 and P4 are presented schematically in Figure 2. When preparing P3 and P4, the polymerization was terminated by diluting the reaction mixture with methanol (1:1 volume ratio) and the diluted reaction mixture was directly used in the spin coating. Given amount of the final polymer solutions were set aside for further polymer isolation and characterization. 

### 2.3. Measurements

**Fourier Transform Infrared Spectroscopy**: Attenuated total reflectance Fourier Transform Infrared (ATRFTIR) spectra were recorded using an IRAffinity-1 spectrophotometer (Shimadzu Company, Kyoto, Japan) equipped with a MIRacleTM ATR accessory (diamond crystal; PIKE Technologies, Madison, WI, USA) providing depth of penetration of the IR beam into the sample of approximately 2 μm. All samples were scanned over a wavenumber range from 4000 to 600 cm^−1^ performing 50 scans at a resolution of 4 cm^−1^.

**Nuclear Magnetic Resonanc**e (NMR) investigations were carried out on a Bruker Avance II + 600 NMR spectrometer (Billerica, MA, USA) using deuterated water or dimethylsulfoxide-d6 as solvent. The copolymer molar mass and/or composition (mole ratio) were calculated based on the integrated proton spectra when comparing the integrals of the signals of PDMA and PEO repeating units.

**Size Exclusion Chromatography** (SEC): The coupled size exclusion chromatography/static light scattering (SEC/SLS) analysis was carried out with an Alliance e2695 Separations Module (Waters Corp., Milford, MA, USA) equipped with Optilab T-rEX refractive index detector and DAWN Heleos II MALS detector (Wyatt Tech., Santa Barbara, CA, USA) Chromatographic separation was achieved by a set of two Waters Ultrahydrogel GPC columns with nominal pore sizes of 120 and 1000 Å. An aqueous solution of 0.1 M NaNO_3_ was used as an eluent at a flow rate of 0.8 mL/min at 35 °C. Polyethylene glycol standards (PSS GmbH) were used for calibration. The data were collected through an e-SAT/IN digital to analog converter and relative molecular mass characteristics (*M*_n_, *M*_w_ and *Ð*) were calculated with the help of Empower 3 software (Waters Corp.).

**Static Light Scattering** (SLS) measurements were carried out on a DAWN HELEOS-II multi-angle laser light scattering (MALLS, Santa Barbara, CA, USA) detector (SLS detectors at 18 angles) equipped with a laser emitting at a wavelength of 658 nm, built-in QELS and COMET ultrasonic cleaning system (Wyatt Tech.). Analyses were performed in a micro batch mode at 25 °C. The specific refractive index increment *dn/dc* was measured at 25 °C on an Optilab T-rEX refractive index detector (Wyatt Tech.) and determined with the Wyatt *dn/dc* software. The data analysis was undertaken using the Rayleigh–Gans–Debye equation (Equation (1)), valid for small interacting particles in the form:

(1) $$\frac{Kc}{R_{\theta }}=\frac{1}{{\bar{M}}_{w}^{agg}}+2A_{2}c$$

where $K\equiv 4\pi^{2}n_{0}^{2}{\left(dn/dc\right)}^{2}/N_{A}\lambda^{4}$ is an optical parameter with $n_{0}^{}$ being the refractive index of toluene; $N_{A}$ is the Avogadro’s constant; λ is the laser wavelength; ${\bar{M}}_{w}^{agg}$ is the aggregate weight average molar mass; $A_{2}$ is the second virial coefficient; *c* is concentration; and, $R_{\theta }$ is the Rayleigh ratio of the polymer solution at a given angle. The absolute weight average molar mass, the radius of gyration, *R_g_*, and the second virial coefficient, *A*_2_, were calculated using Berry or Zimm fit methods processed with ASTRA 6 (Wyatt Tech.) software. All stock solutions (5 g·L^−1^) were prepared directly by dissolving copolymer in bi-distilled water. Different formulations of the nanostructures were obtained by dilution of stock solutions with appropriate amounts of solvent and subsequent vortexing. The stock solutions for the light scattering measurements were purified from dust through polyvinylidene difluoride (PVDF) membranes of 0.45 μm pore size and diluted with a filtered solvent.

### 2.4. Thin Films Preparation

Thin polymer films with thicknesses of 95 nm were prepared by the method of spin coating (3000 rpm, 30 s) on silicon substrates using P1 and P2 polymer solutions in methanol with concentrations of 0.5 wt %. Additionally, by varying the concentration of P1 solution to 0.25 and 1 wt %, films with thicknesses of 55 and 215 nm, respectively, were deposited for comparative purposes. In order to obtain films with good optical quality from polymer P3, a synthesis time of at least 5.5 h was required. After dilution of the initial solution with methanol 5, 7.5 and 10 times, films with thicknesses of 80, 135 and 305 nm were obtained, respectively, for postdeposition annealing of 30 min at 60 °C. When a post deposition annealing of 30 min at 180 °C was applied, thinner films were obtained: 70, 100 and 200 nm, respectively. Thin films from polymer P4 with thicknesses of 60 nm were deposited at synthesis time of 2, 5 and 24 h after dilution of the initial solution with methanol in the ratio 1:1.

### 2.5. Thin films characterization

Refractive index, *n*, extinction coefficient, *k* and thickness, *d*, of the films were calculated from reflectance spectra measured with UV-VIS-NIR spectrophotometer (Cary 5E, Varian, Australia) using a previously developed calculation procedure including two-stage nonlinear curve fitting [25]. The sensing behavior of polymer films was tested by measuring the optical signal (i.e., reflectance at normal light incidence) at different values of relative humidity, %RH. The different levels of humidity in the measuring cell were realized using a homemade bubbler system that generates vapors from liquids [26]. The sensitivity of the sensor is calculated according to Equation (2):

(2) $$S\;=\;\frac{∆R}{∆\%\mathrm{RH}}$$

where Δ*R* is the change of film’s reflectance in % which is provoked by the alteration of relative humidity, Δ%RH.

## 3. Results and Discussions

PDMA copolymers studied in this work were prepared by applying cerium ion initiated redox polymerization in mild and environmentally tolerant reaction conditions: Aqueous media, low reaction temperature, and easy products workup. The method is well known in the literature and widely used in the synthesis of graft copolymers mainly based on poly(vinyl alcohol) and other hydroxyl groups abundant polymers [27,28,29]. Di- and triblock copolymers of PEO based on a cerium ion redox system have also been reported [30,31,32]. The applied ceric ion redox system provides linear block or branched copolymer architectures in an effective one-pot one-stage synthetic procedure depending on the hydroxyl precursor and cross-linker used. Copolymer structure and composition of resulting copolymers were characterized by using conventional spectroscopic methods. Table 1 summarizes the macromolecular characteristics of the copolymers.

**Figure 3:**
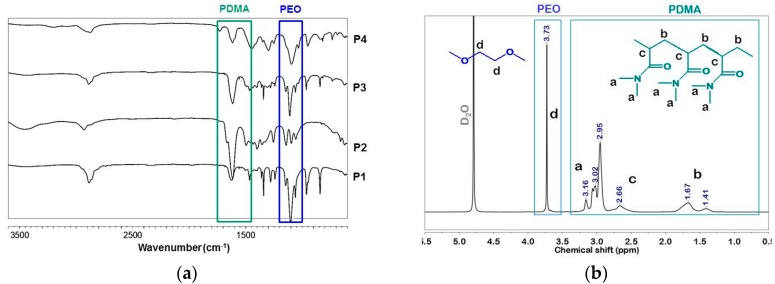
(**a**) Overlaid ATRFTIR spectra of copolymers P1-P4; and (**b**) ^1^H NMR spectrum of P1 (600 MHz; solvent D_2_O).

Figure 3a shows the ATRFTIR spectra of the copolymers obtained where all typical peaks of the expected copolymer constituents are visible: The strong band at 1614 cm^−1^ ascribed to the PDMA amide group and the peak at 1497 cm^−1^ assigned to H–C–H bending vibrations of the methyl group, attached to nitrogen. The band at approximately 1101 cm^−1^, assigned to the characteristic absorption of C–O–C groups, confirms the presence of PEO segments in P1, P3, and P4 copolymers.

The obtained copolymers P1–P4 were further characterized by using NMR spectroscopy. The overlaid proton NMR spectra of copolymers P1–P4 are shown in Figure S1. The compositions of the copolymers were estimated from the integrated ^1^H NMR spectra (Table 1). The molar ratio of PDMA to PEO components was calculated as the ratio of the integrated intensity of the signal at 3.7 ppm assigned to the –CH_2_CH_2_O– protons of PEO to the overall integral of signals at 2.8–3.2 ppm assigned to the –CH_3_ groups of PDMA (Figure 3b). In the case of the linear triblock copolymer, P1, molar mass was calculated based on the known molar mass of PEO2000 and molar ratio of PDMA to PEO segments. Estimation of the molar mass of the star-shaped copolymer, P2, was based on the ratio of the integrated signals of methyl groups from PDMA (2.8–3.2 ppm) to those of the methyl group of functional initiating moiety TMP appearing at 0.7 ppm. Although the proton NMR spectra of P3 and P4 were well resolved (Figure S1) the estimation of the molar masses from NMR was impossible due to the complex macromolecular structure of these copolymers. 

Macromolecular characteristics of the copolymers were determined by SEC. SEC analysis of the linear block copolymer, P1, by using organic eluent such as tetrahydrofuran (THF) showed underestimated molar mass (*M*_n_ 3100 g·mol^−1^; *Ð* 1.07) as compared to the theoretical one, as well as to those determined by NMR (Table 1). We assume that SEC analyses performed in aqueous solution (water-methanol mixture or water containing NaNO_3_) is much more preferable for DMA-based copolymers, despite the partial aggregation that might occur. This was the reason SEC analyses were performed in water as an eluent, which is a good solvent for all studied copolymers. Moreover, copolymers P2, P3, and P4 showed limited or poor solubility in THF.

As seen in Table 1, molar masses of copolymers obtained from aqueous SEC measurements differ substantially from those determined by ^1^H NMR (Table 1). Overlaid SEC traces for two of the obtained copolymers are shown in Figure S2. A possible explanation of the discrepancy between the results from NMR and SEC analyses in case of the linear copolymer, P1, could be that copolymers aggregate in aqueous solution due to intermolecular association, which results in an increase of the hydrodynamic volume of macromolecules and of molecular mass, respectively. The existence of a shoulder at the side of the lower elution time in the chromatogram of the P1 copolymer was attributed to the heterogeneity of the initiator system. In the case of P4, clearly this highly branched product adopts a shrunken conformation due to intramolecular association, which effectively decreases its hydrodynamic volume and shifts the peak to the lower molar mass region. Although it is difficult to estimate quantitatively the contribution of such intramolecular association, the observed overall molar masses are evidently higher than the values determined from ^1^H NMR spectra except for the highly branched P4 copolymer.

Further elucidation of the aggregation phenomena in aqueous solutions of the copolymers was obtained by using SLS. Zimm plots for aggregates formed from the P1 copolymer are presented in Figure S3. There is a noticeable curvature of the angular dependence and a better description of the data is achieved with a second order fit. Non-linearity, although not strictly observed, is anticipated when large aggregates are present, and the product *qR_g_* < 1 (here, *q* is the scattering vector) may no longer hold at higher angles, which supports the assumption of formation of aggregates in aqueous solution already observed by SEC. In contrast, the linear Zimm plot in accordance with first order polynomial fitting observed for the P4 copolymer, implies that an associative process occurs by a different mechanism as indicated by the SEC results presented and discussed above (Table 1 and Figure S3).

In order to study the influence of the different macromolecular architecture of the polymers on their optical and humidity-sensing properties, thin polymer films are deposited by the method of spin coating and films’ properties are investigated. In our previous study, we have shown that the postdeposition annealing at a temperature of 180 °C maximizes the sensitivity of PDMA-*b*-PEO-*b*-PDMA thin films [23]. At this temperature, the change in the film’s refractive index and thickness when humidity changes from 5 to 95% RH is the strongest [23]. These results are obtained with films with thicknesses of 80 nm. However, considering different properties of thin films with different thicknesses, it could be expected the thickness of the films will influence their optical and sensing properties. To check this we prepare thin films from P1 copolymer with thicknesses in the range 55–215 nm and study their optical and sensing properties after postdeposition annealing at 180 °C for 30 min. 

**Figure 4:**
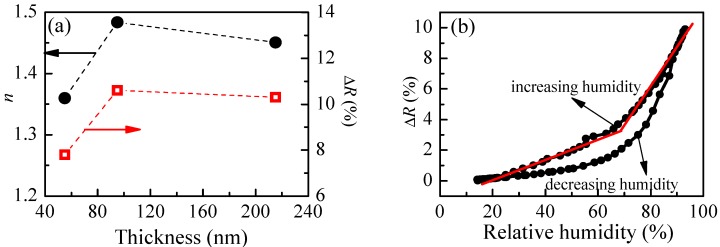
(**a**) Thickness dependence of P1 film’s refractive index, *n*, and reflectance change, Δ*R*, due to a change of relative humidity from 5%RH to 95%RH; and (**b**) Variation of reflectance with humidity of P1 thin film with thickness of 95 nm.

The calculated refractive index of P1 films with different thicknesses and the reflectance change when relative humidity increases from 5%RH to 95%RH are shown in Figure 4a. The smallest value of refractive index (1.36) is obtained for the thinner film. With increasing the film’s thickness, *n* increases and reaches a value of 1.48 for film with thickness of 95 nm. There is a small decrease of *n* (1.45) for the thickest film. Interestingly, the thickness dependence of reflectance change (Δ*R*), when humidity changes from 5%RH to 95%RH, is very similar to the thickness dependence of the refractive index, and we may conclude that there is a relationship between them. Furthermore, it is well known that the refractive index is directly related to the density of material, and the low value of *n* for the thinnest film means that its density is the lowest. Therefore, we could make a hypothesis that the sensitivity of polymer films toward humidity is related to some extent to their density and is higher for denser films. 

When exposed to humidity, the polymer chains swell, resulting in an increase of film thickness and a decrease of refractive index, finally leading to a change of reflectance of the films, because *R* is a function of both parameters. Therefore, if the dependence of reflectance on relative humidity is known, the sensing could be performed by monitoring film reflectance with time. In order to reveal the reflectance versus humidity dependence, we measure *R* at different values of relative humidity in increasing and decreasing humidity modes. The variation of reflectance of P1 film (thickness of 95 nm) with humidity is shown in Figure 4b. Two regions with different slopes (i.e., sensitivity) could be distinguished where the measured signal (i.e., reflectance) changes linearly with humidity. The sensitivity, calculated using Equation (2), is 0.07% in the first humidity region (20–70%RH) and increases substantially to 0.27% for the region of higher humidity (70–95%RH). Considering that the measuring error in reflectance is 0.3%, sensitivity of 0.27% means that by using P1 thin film we could distinguish variation of humidity down to 1.3%RH, because the expected change in signal is 0.35%, which is higher than the measuring error. In the region of lower humidity, the sensor could resolve 5%RH variation in humidity. The disadvantage of using P1 film as an optical humidity sensor is the hysteresis that is observed in decreasing humidity mode. 

The next step of our investigation concerns star-shaped PDMA polymer (P2). In our previous study, when comparing the humidity changes of PDMA thin films to those of corresponding PDMA-PEO di- and triblock copolymers, we have obtained the strongest changes in refractive index and thickness for PDMA homopolymer thin films [23]. Considering this together with the star-shaped macromolecular structure of P2, an enhancement of sensing properties for P2 films could be expected. Thin films of P2 polymer with thickness of 92 nm are prepared by spin coating and their refractive index is calculated using a previously developed two-step procedure of nonlinear curve fitting [25]. The refractive index of P2 films at wavelength of 600 nm is determined to be 1.48, which matches exactly the value of *n* for P1 film with a similar thickness (95 nm). 

**Figure 5:**
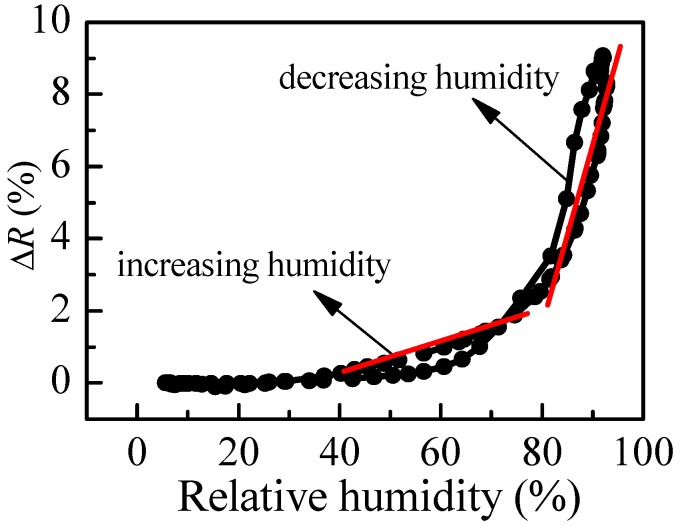
Variation of reflectance of P2 thin film with thickness of 92 nm with humidity.

The variation of reflectance of P2 film with humidity is presented in Figure 5. It is seen that the values of *R* in the increasing mode of humidity are very close to the values for the decreasing mode, so the hysteresis is very weak. This is a very important characteristics of a sensor and could be regarded as a good prerequisite for using P2 polymer as an active medium for humidity sensing. In addition, two linear ranges for humidity higher than 40%RH are observed. The calculated sensitivity is 0.05% in the 40–75%RH range and increases to 0.46% for RH values higher than 80%RH. Therefore, for RH higher than 80%RH, the P2 thin film could resolve less than 1%RH. However, the P2 film is insensitive for relative humidity less than 40%RH, because no change in the measured signal is obtained in this humidity range. 

The comparison of humidity sensing abilities of P1 and P2 (Figure 4 and Figure 5) shows that P1 films are more suitable for optical humidity sensing, because a linear variation of reflectance is obtained for a wide range of humidity, while P2 films are insensitive up to 40%RH. To increase further the sensitivity and to decrease the hysteresis level, thin films from the two synthesized branched copolymers (P3 and P4) are studied.

**Figure 6:**
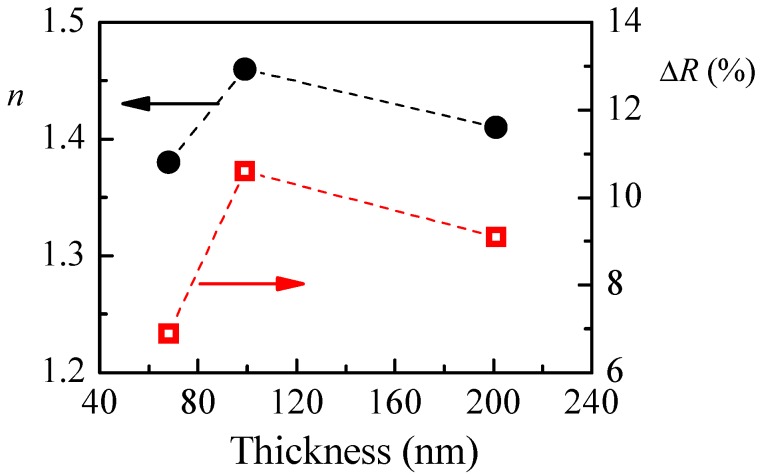
Thickness dependence of P3 film’s refractive index, *n*, and reflectance change, ΔR, due to a change of relative humidity from 5 to 95%RH.

Thin films from P3 with different thicknesses are deposited on silicon substrate by the method of spin coating. The films are annealed at 60 and 180 °C, and their optical constants (*n* and *k*) and thicknesses are determined from their reflectance spectra. Further, reflectance spectra of P3 polymer films with thickness in the range 70–200 nm are measured at low (5%RH) and high (95%RH) humidity levels. Reflectance change and refractive index are presented in Figure 6 as a function of films thickness. Similar to the case for P1 films, for P3 films the highest sensitivity towards humidity is obtained for film with the highest refractive index. Furthermore, the obtained humidity responses of 6.9%, 10.6% and 9.1% for P3 polymer films with thicknesses of 68, 99 and 201 nm, respectively, are very close to the values for P1 films: 7.8%, 10.6% and 10.3% for thicknesses of 55, 95 and 215 nm, respectively.

**Figure 7:**
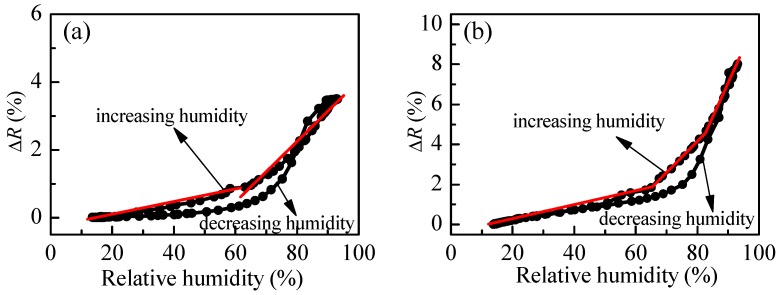
Variation of reflectance with humidity for P3 thin films annealed at 60 °C (**a**) and 180 °C (**b**) for 30 min after deposition.

The variation of reflectance of P3 films with humidity is presented in Figure 7 for postdeposition annealing of 60 and 180 °C. Generally, the sensitivity increases with annealing temperature. In the first case (60 °C) two linear ranges could be distinguished having different sensitivities. In the 10–60%RH range, S is 0.02% and increases slightly to 0.08% for relative humidity higher than 60%RH. In the case of higher thermal treatment of the polymer film, three linear regions are detected with increasing sensitivity: 0.04% for 10–65%RH; 0.18% for 65–80%RH; and, 0.25% for RH values higher than 80%RH. The comparison between Figure 4b and Figure 7 confirms our expectation of hysteresis improvement in the case of branched polymer P3. It is seen that the difference between reflectance values for increasing and decreasing humidity modes substantially decreases for P3 film as compared to linear polymer film P1. Moreover, for relative humidity less than 60% and higher than 85%, there is no hysteresis. To improve further the sensing capabilities, we synthesize another branched polymer, P4, which consists of a loosely cross-linked macromolecular structure due to the trifunctional cross-linker TMPTA used. 

**Figure 8:**
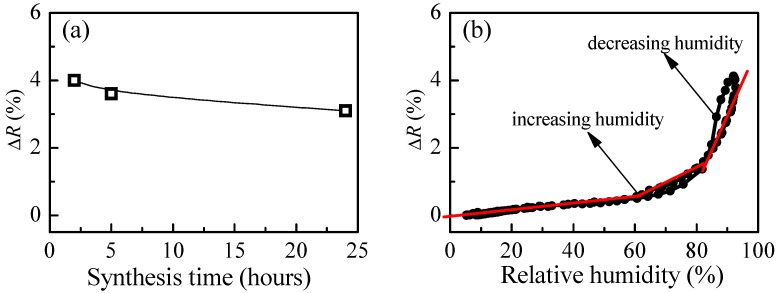
(**a**) Reflectance change, ΔR, due to a change of relative humidity from 5%RH to 95%RH as a function of synthesis time for P4 films; and (**b**) variation of reflectance with humidity for P4 thin films.

Figure 8a presents the change of reflectance of P4 film when exposed from low to high humidity levels as a function of synthesis time. The strongest change is obtained after 2 h of synthesis. With increasing the time of synthesis, a slight decrease of the measured signal is detected. Our additional DLS (Dynamic Light Scattering) measurements have shown an increase of polymer particles with time that could be the possible reason for the observed deterioration in sensing properties. The calculated values for refractive index are in the range 1.40–1.41, and they are independent of the synthesis time.

The variation of measured signal (reflectance) with relative humidity for P4 thin films is presented in Figure 8b. The match between reflectance values for both humidity modes (increasing and decreasing) is very good (i.e., the hysteresis is very weak). Moreover, compared to branched polymer P3, a substantial improvement takes place. Three linear ranges in the R-RH dependence could be discriminated. In the first (5–60%RH), the sensitivity is very low (i.e., 0.01%), then increases to 0.05% in the 60–80%RH range and reaches a value of 0.17% for relative humidity higher than 80%RH.

The comparison between Figure 7b and Figure 8b shows that although the hysteresis is smaller in the case of loosely cross-linked P4, branched polymer P3 is more suitable for optical sensing of humidity because its average overall sensitivity is 0.16%, while sensitivity of P4 is half that of P3 (0.08%).

Table 2 summarizes the values of refractive index at a wavelength of 600 nm, sensitivities calculated using Equation (2), and the resolution in relative humidity that is the lowest that could be measured by the sensor using P1–P4 thin films as sensitive media.

## 4. Conclusions

In this work, poly(*N*,*N*-dimethylacrylamide)-based copolymers were shown to be advantageous new materials in designing thin polymer films for optical sensing of humidity. Ceric ion redox polymerization used for the synthesis of copolymers provided diverse linear block or branched copolymer architectures in effective one-pot one-stage reaction procedures in mild and environmentally tolerant reaction conditions (i.e., aqueous polymerization at low temperature). It was demonstrated that the chemical structure and composition, as well as macromolecular architecture of the copolymers plays a crucial role in their optical and humidity-sensing behavior. Macromolecular structure influences both the sensitivity and hysteresis level. It is demonstrated that the most suitable polymer for optical sensing of humidity is the branched polymer, P3, in which the optimal balance between sensitivity and hysteresis is achieved. The highest sensitivity is obtained for RH > 80%RH, where humidity is measured with a resolution of 1.4%. For a relative humidity range of 65–80%RH, 2% of humidity could be resolved, while for humidity less than 65%RH, the measurement resolution is 8.8%. All films have thicknesses less than 100 nm, which provides a fast and reversible response to humidity changes and guarantees sustained humidity-sensing properties.

## Figures and Tables

**Table 1:** Macromolecular characteristics of the developed linear and branched copolymers.

Code	Copolymer	PDMA:PEO Mole Ratio (NMR)	*M*_n_, g·mol^−1^ (NMR)	*M*_n_, g·mol^−1^ (SEC)	*Ð* (SEC)
**P1**	PDMA-*b*-PEO-*b*-PDMA	2.5	13,200	763,600	1.59
**P2**	TMP-[PDMA]_3_	-	5500	20,000	1.96
**P3**	[PDMA-*b*-PEO-*b*-PDMA]-*cross*-PEGDA	1.7	-	107,000	2.90
**P4**	[PDMA-*b*-PEO-*b*-PDMA]-*cross-*TMPTA	0.7	-	2900	1.06

**Table 2:** Refractive index (*n*), sensitivity and resolution of thin films of P1–P4 polymers.

Code	*n* at 600 nm	Sensitivity (%/%RH)	Resolution (%RH)
**P1**	1.48	0.07 (20–70%RH)	5
		0.27 (70–95%RH)	1.1
**P2**	1.48	0.05 (40–75%RH)	7
		0.46 (80–95%RH)	0.8
**P3**	1.46	0.04 (10–65%RH)	8.8
		0.18 (65–80%RH)	2
**P4**	1.41	0.25 (80–95%RH)	1.4
		0.01 (5–60%RH)	35
		0.05 (60–80%RH)	7
		0.17 (80–95%RH)	2.1

## Supplementary Materials

The following are available online at http://www.mdpi.com/2073-4360/10/7/769/s1, Figure S1: Overlaid ^1^H NMR spectra (600 MHz; solvent D_2_O) of copolymers P1–P4; Figure S2: Overlaid SEC traces of linear copolymer P1 and branched copolymer P4; Figure S3: Zimm diagrams for copolymers P1 (left) and P4 (right).
