# Supplementary material for: Influence of Macromolecular Architecture on the Optical and Humidity-Sensing Properties of Poly(N,N-Dimethylacrylamide)-Based Block Copolymers
Source: Polymers (Basel). 2018 Jul 13;10(7):769. doi: 10.3390/polym10070769 (PMC6403531; doi:10.3390/polym10070769)
Supplement: Supplementary file 1 [file polymers-10-00769-s001.pdf]

polymers

MDPI

Supplementary Material

Influence of macromolecular architecture on the optical and humidity sensing properties of poly(N,N-dimethylacrylamide)-based block copolymers

Katerina Lazarova [1], Marina Vasileva [1], Sijka Ivanova [2], Christo Novakov [2], Darinka Christova [2] and Tsvetanka Babeva [1]

[1] Institute of Optical Materials and Technologies ''Acad. J. Malinowski'', Bulgarian Academy of Sciences, Acad. G. Bonchev str., bl. 109, 1113 Sofia, Bulgaria; babeva@iomt.bas.bg
[2] Institute of Polymers, Bulgarian Academy of Sciences, Acad. G. Bonchev Str., bl. 103-A, 1113 Sofia, Bulgaria; dchristo@polymer.bas.bg

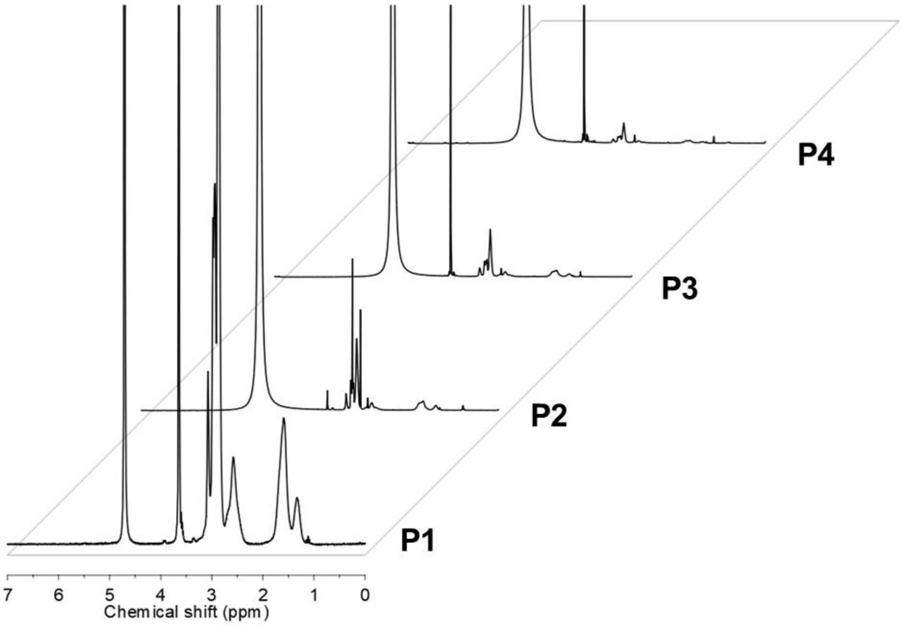

Figure S1. Overlaid ^{1}H NMR spectra (600 MHz; solvent D_2O) of copolymers P1-P4.

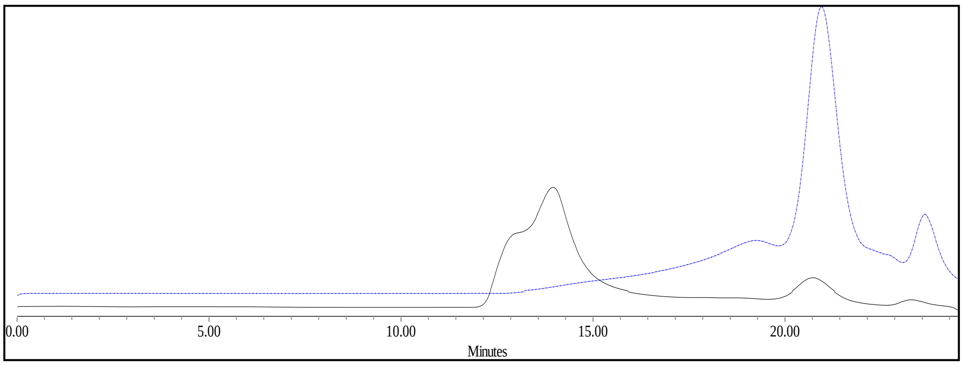

Figure S2. Overlaid SEC traces of linear copolymer P1 (black) and branched copolymer P4 (blue).

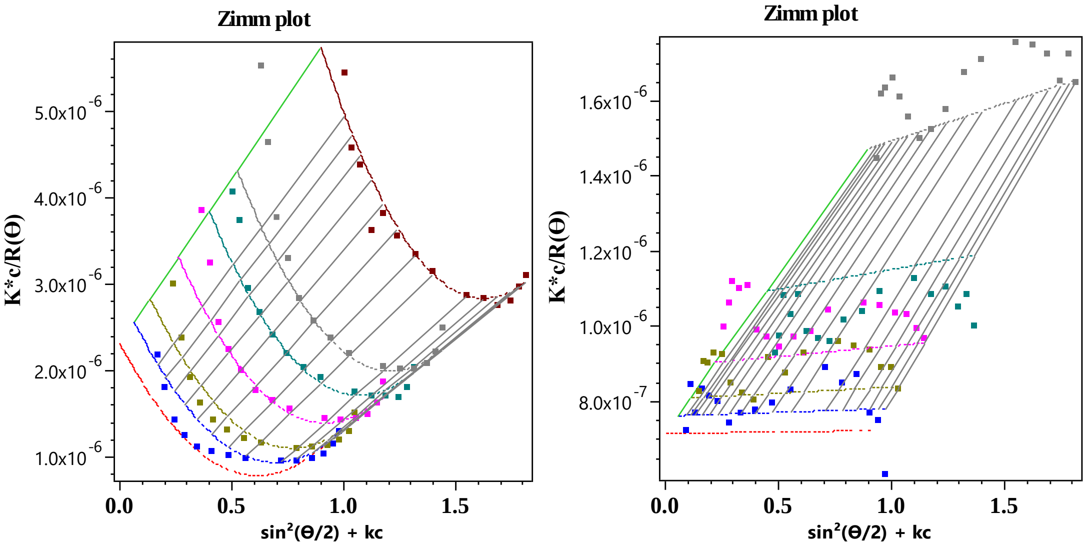

Figure S3.Zimm diagrams for copolymers P1(left) and P4 (right).
